# Genomic and phenotypic characterization of the species *Acinetobacter venetianus*

**DOI:** 10.1038/srep21985

**Published:** 2016-02-23

**Authors:** Marco Fondi, Isabel Maida, Elena Perrin, Valerio Orlandini, Laura La Torre, Emanuele Bosi, Andrea Negroni, Giulio Zanaroli, Fabio Fava, Francesca Decorosi, Luciana Giovannetti, Carlo Viti, Mario Vaneechoutte, Lenie Dijkshoorn, Renato Fani

**Affiliations:** 1 Dep. of Biology, ComBo, Florence Computational Biology Group, University of Florence, I-50019 Sesto F.no, Italy; 2 Dep. of Biology, LEMM, Laboratory of Microbial and Molecular Evolution Florence, University of Florence, I-50019 Sesto F.no, Italy; 3 Dep. of Clinical and Experimental Biomedical Science “Mario Serio”, University of Florence, Viale Pieraccini, 6, I-50139 Florence, Italy; 4 Dep. of Civil, Chemical, Environmental and Materials Engineering (DICAM), University of Bologna, I-40131 Bologna, Italy; 5 Dipartimento di Scienze delle Produzioni Agroalimentari e dell’Ambiente (DISPAA), University of Florence, P.le delle Cascine, 24, Florence, 50144, Italy; 6 Laboratory Bacteriology Research, Faculty Medicine & Health Sciences, University of Ghent, Belgium; 7 Dep. of Infectious Diseases, Leiden University Medical Center, PO Box 9600, 2300 RC, Leiden, The Netherlands

## Abstract

Crude oil is a complex mixture of hydrocarbons and other organic compounds that can produce serious environmental problems and whose removal is highly demanding in terms of human and technological resources. The potential use of microbes as bioremediation agents is one of the most promising fields in this area. Members of the species *Acinetobacter venetianus* have been previously characterized for their capability to degrade *n*-alkanes and thus may represent interesting model systems to implement this process. Although a preliminary experimental characterization of the overall hydrocarbon degradation capability has been performed for five of them, to date, the genetic/genomic features underlying such molecular processes have not been identified. Here we have integrated genomic and phenotypic information for six *A. venetianus* strains, i.e. VE-C3, RAG-1^T^, LUH 13518, LUH 7437, LUH 5627 and LUH 8758. Besides providing a thorough description of the *A. venetianus* species, these data were exploited to infer the genetic features (presence/absence patterns of genes) and the short-term evolutionary events possibly responsible for the variability in *n*-alkane degradation efficiency of these strains, including the mechanisms of interaction with the fuel droplet and the subsequent catabolism of this pollutant.

Representatives of the genus *Acinetobacter* are Gram-negative bacteria, aerobic, non-fermenting, non-fastidious, catalase-positive, oxidase-negative and have a DNA GC content of 34.9–47%^1^. Members of this genus can be isolated from a broad range of different environments, including water, soil and living organisms and, despite having been described as non-motile, possess different forms of motility (e.g. twitching^2^). Importantly, this genus also features both opportunistic pathogens primarily associated with hospital-acquired infections (*Acinetobacter baumannii*, *A. nosocomialis* and *A. pittii*) and biotechnologically relevant strains to be employed, for example, in synthetic biology pipelines (*A. baylyi* ADP1).

Up to now, most research on this genus has focused on clinical isolates and, in particular, those belonging to the species *A. baumannii*. These microorganisms have shown a remarkable ability to acquire antibiotic resistance genes (also by means of horizontal gene transfer, HGT^3^) and infections by particular strains are now close to being untreatable^4^,^5^. Nevertheless, other *Acinetobacter* species have started attracting increasing attention in both environmental and biotechnological areas. Indeed, some *Acinetobacter* representatives are known to be involved in biodegradation of a number of contaminants such as halogens^6^, phenols^7^, crude oil^8^,^9^. Remarkably, over the last decades various strains belonging to this genus have been demonstrated to be also involved in alkane degradation and to be capable of thriving on these highly reduced organic compounds^9^,^10^,^11^. The use of such strains for bioremediation of alkane-polluted sites might provide valuable advances to solve this environmental issue.

The *A.venetianus* species has been described in 2009^12^ and includes marine hydrocarbon-degrading strains. It has been proposed as an interesting model system for studying the mechanisms underlying the process of alkane degradation and, more in general, a valuable platform for bioremediation of contaminated areas^13^,^14^.

Interestingly, different strains of *A. venetianus* have been shown to exploit diverse strategies to perform alkane degradation, starting from the adhesion to the diesel fuel drops. In *A. venetianus* VE-C3, for example, the interaction between the diesel fuel droplets and the cell envelope is a complex process during which *n*-alkanes induce glycosylation of membrane proteins involved in oil uptake^15^ followed by internalization of diesel fuel droplets and catabolism of its constituents^16^. A different strategy is adopted by *A. venetianus* RAG-1^T^, whose cells can produce a strong biosurfactant-the lipopolysaccharide emulsan-that interfaces between cell membranes and oil^17^,^18^,^19^,^20^, thus facilitating the process of *n-*alkanes uptake. The latter strain has gained attention in the last decades given its bioremediation potential and the finding that emulsan might be employed as an adjuvant of the immune system response, being able to stimulate macrophages thanks to its chemical structure^21^.

A considerable body of data has been produced so far for some *A. venetianus* members. The genome sequence of two representatives (strains VE-C3 and RAG1^T^) has been determined and analysed^22^,^23^. *A. venetianus* VE-C3 was shown to possess a wide range of determinants whose molecular functions are probably related to the survival in a strongly impacted ecological niche, including genes involved in the metabolism of *n*-alkanes and in the resistance to toxic metals (e.g. arsenic, cadmium, cobalt and zinc). Also, the importance of HGT in shaping the genome of this species was inferred from its relatively high content of genes involved in DNA mobilization and the presence of compositionally atypical chromosome regions. The genome sequence of strain RAG1^T^, instead, contributed to define the set of *alk*-like genes possessed by this microorganism, together with other genes possibly involved in alkane degradation and tolerance to highly polluted ecological niches.

Previously, Mara *et al*. (2012) performed a molecular and phenotypic characterization of a set of *Acinetobacter* strains (including some *A. venetianus* representatives). This study shed some light on the overall strategies adopted by *Acinetobacter* strains with regard to diesel fuel degradation, suggesting a higher efficiency in this process by *A. venetianus* members relative to the other microorganisms tested. Also, a large variability in the efficiency of *n*-alkane degradation among *A. venetianus* strains was observed, with some strains (e.g. RAG-1^T^) clearly outperforming all the others. This is somehow surprising, given the phylogenetic proximity of these strains but, on the other hand, raises the opportunity to investigate in great details the underlying genetic bases of the phenotypic diversity concerning the different strategies/abilities in hydrocarbon degradation. Indeed, in principle, comparing genomes of closely related microorganisms when searching for genetic variations responsible for different phenotypes should lead to clear and testable findings; this is not always straightforward when comparative analyses are performed on the genome of distantly related and phenotypically unrelated microbes.

Here, we obtained and integrated genomic and large-scale phenotypic information from six *A. venetianus* strains, i.e. VE-C3, RAG-1^T^, LUH 13518, LUH 7437, LUH 5627 and LUH 8758. These data were used to infer the genetic features (genes presence/absence patterns) and the short-term evolutionary events possibly related to the variability in *n*-alkane degradation efficiency within this species, including the mechanisms of interaction with the fuel droplet and the subsequent catabolism of this pollutant.

Results obtained allowed the identification of strain-specific differences in the overall strategy of alkane degradation and possibly responsible for higher (and lower) efficiencies in this process. More in general, this study represents an example of how multi-omics information can be used for connecting microbial phenotype(s) to their underlying genotype(s).

## Results

### Hydrocarbon degradation capabilities

**Figure 1:**
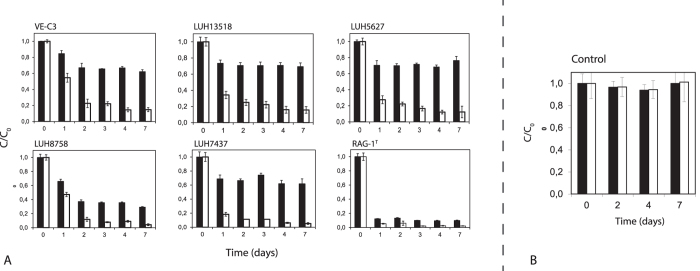
Diesel fuel degradation capabilities over the course of one week for the six *A. venetianus* strains tested. Residual over initial HC concentration (C/C_0_) is plotted over time for each strain (**a**) and for the abiotic control (**b**). Black bars represent total Diesel fuel hydrocarbons and white bars the sum of C_10_ to C_25_ *n*-alkanes.

Diesel fuels are complex mixtures of hydrocarbons composed for about 75% of saturated hydrocarbons (primarily *n*-, iso- and cycloalkanes), and for about 25% of aromatic hydrocarbons (including naphthalenes and alkylbenzenes). We specifically addressed the degradation capabilities of each of the six *A. venetianus* strains (namely VE-C3, RAG-1^T^, LUH 13518, LUH 7437, LUH 5627 and LUH 8758) towards diesel fuel total hydrocarbons as well as their main fraction, i.e., *n*-alkanes. Regarding the degradation of diesel fuel total hydrocarbons (black bars in Fig. 1), strains VE-C3, LUH 13518, 5627 and LUH 7437 degraded less than 40% of the initial amount, while LUH 8758 and RAG-1^T^ caused noteworthy depletions at the end of incubation (71 ± 1% and 90 ± 1%, respectively). With regard to the degradation of C10–C25 *n*-alkanes (white bars in Fig. 1), 85 ± 3%, 84 ± 4% and 88 ± 7% biodegradation was attained by strains VE-C3, LUH 13518 and 5627 at the end of the incubation, whereas remarkably higher degradation extent was reached by the other strains (biodegradations of 96 ± 1%, 95 ± 1% and 98 ± 0.3% for LUH 8758, LUH 7437 and RAG-1^T^, respectively, at the end of the incubation). Among the latter strains, differences were observed in the degradation rate of *n*-alkanes, being RAG-1^T^ the faster, followed by LUH 7437 and LUH 8758 (biodegradations after 1 day of incubation of 95 ± 0.4%, 82 ± 6% and 53 ± 3%, respectively). The remarkable hydrocarbons degradation capability of strain RAG-1^T^ is consistent with and complements previous studies reporting that growth of strain RAG-1^T^ on crude oil or hexadecane is completed after 1–1.5 days^24^,^25^. The degradation efficiency for *n*-alkanes having different molecular weight (MW) was also evaluated (Supplementary Figure S1). Most of the strains displayed a preferential degradation of low MW *n*-alkanes, with the degradation efficiency inversely correlated to the hydrocarbon MW, with the exception of RAG-1^T^ strain, which almost completely depleted all C10-C25 *n*-alkanes regardless of their MW.

Overall, results obtained (shown in Fig. 1) revealed that the six *A. venetianus* strains are capable of degrading diesel fuel hydrocarbons and, more extensively, the *n*-alkane fraction (white bars in Fig. 1), albeit with a rather large variability among them in terms of extent and rates. In conclusion, taking into consideration both the final extent and rate of degradation of both total hydrocarbons and *n*-alkanes carried out by each strain (see Materials and Methods), we established the following ranking for the six *A. venetianus* strains, from the most to the less efficient: RAG-1^T^ > LUH 8758 > LUH 7437 > LUH 5627 > LUH 13518 > VE-C3. Despite the better overall ranking of strain LUH 8758 compared to LUH 7437, the two strains behave remarkably differently in terms biodegradation efficiency of *n*-alkanes and total diesel hydrocarbons: while the former degrades total hydrocarbons to a much higher final extent, the latter degrades remarkably faster *n*-alkanes. This indicates that LUH 7437 is a more efficient *n*-alkane degrader, whereas strain LUH 8758 is most likely more effective in the biodegradation of other fractions of diesel fuel hydrocarbons (other saturated hydrocarbons such as iso- or cycloalkanes, for instance), that were not specifically monitored.

### Phenotype Microarray data analysis

**Figure 2:**
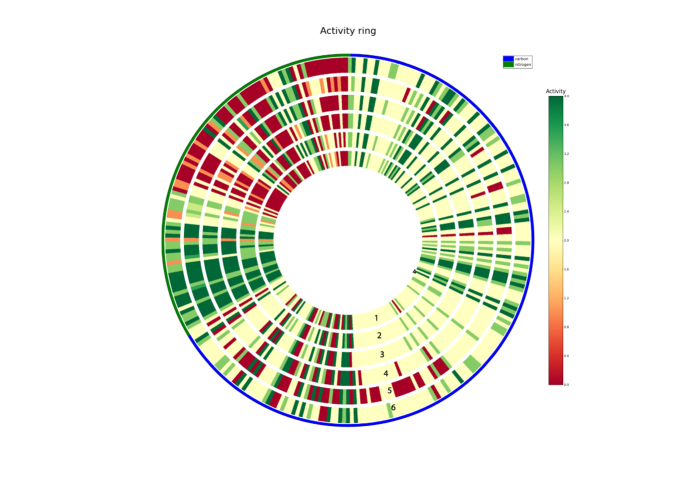
Phenotype microarray experiment. Circular plot representing the different growth phenotypes of the six *A. venetianus* strains as assessed by the PM experiment (1 = LUH 13518, 2 = LUH 5627, 3 = LUH 7437, 4 = LUH 8758, 5 = RAG-1^T^, 6 = VE-C3). Each concentric circle represents one of the six strains whereas each radial strip corresponds to a single tested phenotype). Each strip is coloured according to the calculated Activity Index (AV) and accounting for the observed growth phenotype of each strain on that specific carbon/nitrogen source. See^27^ for details on the AV calculation.

In order to understand whether this considerable metabolic diversity among the six *A. venetianus* strains is limited to the hydrocarbon degradation process or, rather, is a more general feature, we analysed in more detail their global metabolic landscape. Phenotype microarray (PM) data previous experiments^26^ were more comprehensively analysed exploiting the recently developed DuctApe suite^27^. Additionally, PM data were *de novo* generated for one of the *A. venetianus* strains, i.e. LUH 13518, that had been excluded from previous experiments. All PM raw data are available as Supplementary Material (Supplementary Dataset S1). Results of these analyses are reported in Fig. 2 in the form of a circular plot. In this figure, each concentric circle represents one of the six strains whereas each radial strip corresponds to a single PM curve (i.e. a tested phenotype). Each strip is coloured according to the calculated Activity Index (AV) and accounting for the observed growth phenotype of each strain on that specific carbon/nitrogen source. Briefly, the AV has a value between 0 and a user defined value k (k > 0), which indicates those wells that exhibit low or no metabolic activity (0) and those with higher metabolic activity (k) within that particular experiment. The AV parameter is calculated through a k-means clustering (with k clusters) on five growth curve parameters (max, area, average height, lag time, and slope). More details on the calculation of the AV can be found in Galardini *et al*. (2014).

This comprehensive analysis revealed an overall phenotypic similarity among all the six strains. Indeed, for most of the carbon and nitrogen sources, the six strains displayed an overlapping trend of growth/no-growth. Nevertheless, a few strain specific peculiarities were observed. *A. venetianus* VE-C3, for example, displayed the lowest AV on several nitrogen sources, revealing its tendency to grow slower on these substrates relative to the other *A. venetianus* strains tested. These N-sources included two dipeptides (Ala-Asp and Ala-Glu), D-alanine, D-lactamide, biuret, tyrosine and tyramine. Similarly, the growth of *A. venetianus* RAG-1^T^ is impaired relative to other *A. venetianus* representatives on specific carbon sources, which included citramalic acid γ-hydroxybutyric acid, quinic and mucic acids, α-methyl-D-mannoside and L-isoleucine. Other strain-specific metabolic capabilities included, for example, the catabolism of sebacic and itaconic acids, D-alanine, γ-amino-N-butyric acid among carbon sources and xanthine, allanthoine and inosine among nitrogen sources (see Supplementary Figure S2). However, when the gene annotations of these genomes were searched for determinants possibly involved in such metabolic processes (and able to explain the observed phenotypic differences) no hints could be derived. This, in turn, suggests that i) either the enzymatic complexes responsible for the different growth phenotype are still unknown/unexplored or ii) other cellular processes are responsible for determining the growth of some of the tested strains on such media (e.g. gene regulation).

Finally, Biolog results match those reported by Vaneechoutte *et al*. (2009) who preliminary described this species phenotypically, despite not all the compounds tested by Vaneechoutte *et al*. were present in our large scale phenotypic test. Indeed, the six *A. venetianus* strains were capable of growing on acetate, 4-aminobutyrate, L-arginine, citrate, L-histidine, L-leucine, D-malate, malonate and showed no (or very reduced) growth on 2,3-butanediol, citraconate, D-glucose, D-gluconate, 4-hydroxybenzoate, DL-lactate, L-ornithine, L-phenylalanine, putrescine, L-tartrate and tricarballylate.

### HC-degrading strains form a consistent cluster in the species tree

Genome sequences for strains VE-C3 and RAG1^T^ had already been determined previously, whereas the genomes of the remaining four strains was *de novo* sequenced and assembled as described in Material and Methods. An overview of the genomic data used for this work is presented in Table 1.

**Figure 3:**
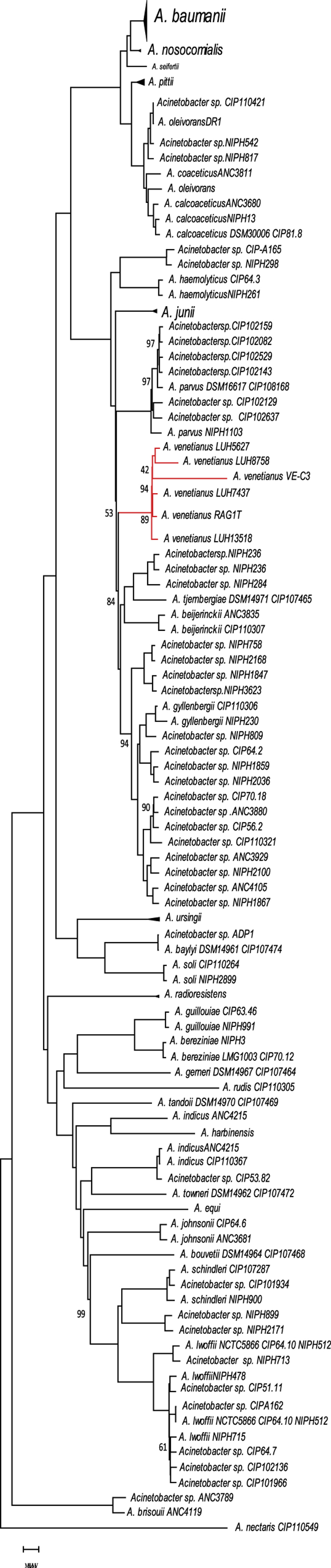
Phylogenetic tree of *Acinetobacter* genus. A genome-scale based phylogeny was reconstructed according to the *Acinetobacter* pangenome. Only bootstrap values lower that 100 are reported. A. venetianus branches are highlighted in red.

First, we checked the phylogenetic coherence among the analysed *A. venetianus* strains and their relationships with the other representatives of the genus *Acinetobacter*, whose genome had been already sequenced using a comprehensive dataset of 143 genomes (see Supplementary Table S1 for the list of genome accessions). The (753) groups of orthologs shared by all the genomes were separately aligned and then concatenated in a single multialignment (embedding 265354 residues). This was then used for the construction (see Material and Methods) of the phylogenetic tree shown in Fig. 3 (see Supplementary Dataset S2 for the original, newick-encoded version of the tree). The analysis of the phylogenetic tree reveals that the *A. venetianus* strains form a strongly supported monophyletic cluster in the same larger clade that also comprises representatives of *A. junii*, *A. beijerinckii* and *A. gyllenbergii*. Furthermore, the observed branching order is in overall agreement with the phylogenetic structure of the genus *Acinetobacter*, recently proposed by Touchon *et al*. (2014)^28^.

### The pangenome of HC degradation

**Figure 4:**
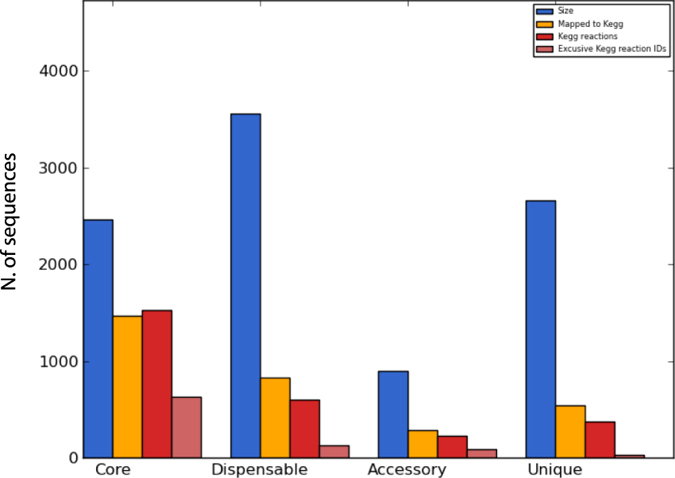
Overall pangenome statistics of the six *A. venetianus* strains. The number of genes mapped to KEGG database (dark yellow), the overall number of KEGG reactions (red) and the number of exclusive KEGG reaction IDs (pink) are reported.

Despite being phylogenetically related, the *A. venetianus* strains show a relatively high divergence in terms of their gene content. Indeed, the structure of the *A. venetianus* pangenome (Fig. 4) revealed that the majority of the genes of the *A. venetianus* pangenome belong to the dispensable genome (3560) whereas only 2462 genes are shared by all the strains, being part of the core genome. Out of the 3560 dispensable genes, 893 comprise the so-called accessory genome, i.e. they are shared by two or more strains, whereas 2667 represent the pool of the unique genome, i.e. they are strain specific genes. The number of unique genes varies among the different strains, ranging from 220 in the case of *A. venetianus* RAG1^T^ to 764 for *A. venetianus* LUH 7437. A closer inspection of the functional content of the core gene repertoire revealed, as might be expected, an enrichment of housekeeping functions, such as cell division and chromosome partitioning, nucleotide metabolism and transport, translation and transcription (data not shown). Instead, analysis of the gene content of the unique and accessory pools revealed interesting strain specific features; the next sections will focus on these differences and their possible effect on the phenotypes (i.e. *n*-alkanes degradation).

### Variability in gene clusters related to adhesion to fuel oil

The construction of the *A. venetianus* pangenome offers the opportunity to investigate the genotypic mechanisms that are most likely responsible of the phenotypic diversity observed among these strains, i.e. the different efficiency in hydrocarbon degradation. Schematically, alkane degradation can be divided into two main stages, i.e. 1) the adhesion of the microbial cell to the hydrophobic fuel oil drops, and its further internalization and 2) the metabolic reactions leading to its degradation. Therefore, we searched the six *A. venetianus* genomes for genetic determinants involved in the corresponding molecular processes.

**Figure 5:**
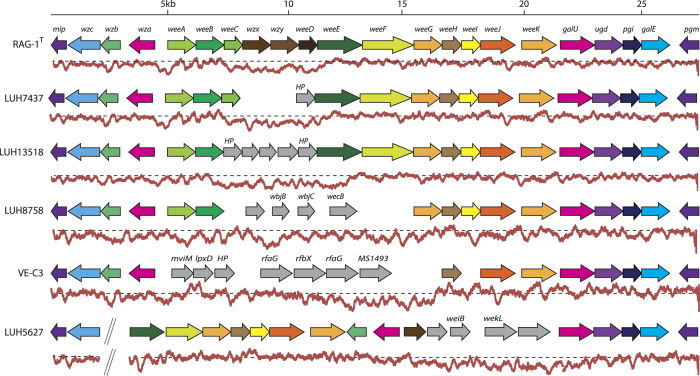
Structure and composition of the emulsan gene cluster in the six *A. venetianus* strains. Below each gene cluster, the average GC content over a 100-bp sliding window is reported (red line) and compared to the average GC content of the same genome (dashed line). Genes extraneous to the reference *A. venetianus* RAG-1^T^ gene cluster are filled in grey and named according to their closest homolog in NCBI nr database (if any).

The process of adhesion to *n*-alkanes has been well characterized in *A. venetianus* RAG-1^T^, which has been shown to produce an extracellular anionic lipoheteropolysaccharide, known as emulsan, to assist in the capture and transport of the carbon sources to the cell^17^,^29^,^30^. A 27 kbp cluster of genes, responsible for the biosynthesis of this amphipathic, polysaccharide bioemulsifier from the oil-degrading *A. venetianus* RAG1^T^, was identified characterized^31^. Here, we explored more thoroughly the distribution and conservation of this cluster in the available *A. venetianus* genomes. Results of this analysis are reported in Fig. 5. As shown, none of the strains shares the same structure of the emulsan cluster and, consequently, this cluster appears to be characteristic of strain *A. venetianus* RAG1^T^. Among all the others, strain LUH 7437 is the one possessing the *wee* gene cluster that resembles most the one of *A. venetianus* RAG-1^T^ with 19 genes with significant sequence similarity shared by the two gene clusters. The genes that are missing (or replaced by genes annotated as hypothetical proteins) are those involved in the polymerization and extrusion of the emulsan molecule, namely *wzx* (putative emulsan repeating unit flippase), *wzy* (putative emulsan repeating unit) and *weeD* (putative glycosyltransferase). Other *A. venetianus* strains (namely LUH 13518, LUH 8758 and VE-C3) display a less conserved structure of this cluster (Fig. 5). Finally, in *A. venetianus* LUH 5726, the emulsan biosynthesis-like genes are spread over a larger chromosome region (35 kb) that also embeds genes apparently unrelated with the biosynthesis of a surfactant-like polymer.

The variability of the emulsan biosynthetic cluster retrieved in the six *A. venetianus* strains analysed provides a possible explanation of the phenotypic differences observed in previous studies. Indeed, besides RAG-1^T^, strain LUH 7437 is the only other strain that was shown to possess emulsifying activity^26^. Remarkably, RAG-1^T^ and LUH 7437 are those strains whose emulsan biosynthetic clusters display the highest conservation among the *A. venetianus* representatives. This, in turn, suggests that the cluster possessed by *A. venetianus*, despite lacking some genes involved in the polymerization/extrusion of the final molecule, may still be able to synthesize an active compound conferring the strain a good efficiency in the degradation of the linear fraction of fuel oil (see Supplementary Figure S1). When searching for possible homologs of the missing genes in the LUH 7437 emulsan cluster (namely *wzx, wzy,* and *weeD*) we did not find any candidate to carry out the original function of the RAG-1^T^ emulsan cluster. This suggests that (still) unknown genes are probably performing polymerization and extrusion of an emulsan-like molecule in LUH 7437. All the other strains did not reveal emulsifying capabilities^26^, in line with an increasing number of missing genes relative to RAG-1^T^ and LUH 7437 clusters. The fact that LUH 13518 only lacks the *weeC* gene compared to LUH 7437 may tentatively suggest a crucial role of this gene in conferring emulsifying capabilities to *A. venetianus* strains.

From an evolutionary viewpoint, it is interesting to note that the variability of the *wee* gene cluster mainly affects the central portion of this chromosome region, whereas both the initial and the final parts of the cluster are conserved across all the strains (with the partial exception of the LUH 5726 strain). Moreover, an analysis on the composition of the DNA regions embedding this cluster in the different strains revealed, in most cases, a notable deviation from the average GC content of the rest of the genome (Fig. 5). In addition to this, the fact that the strain-specific genes embedded in the central part of each of the emulsan clusters analysed (the grey arrows in Fig. 5) have no homologs in the other *A. venetianus* genomes, suggests that the extant structure of the emulsan biosynthetic cluster in these strains may be the result of multiple (independent) HGT events that led to the gain/loss of genes involved in this metabolic pathway. The phenotypic effect of such modifications is reflected by the capability to emulsify fuel oil in some strains (namely RAG-1^T^ and LUH 7437) and the absence of such feature in the others (LUH 8758, LUH 13518, VE-C3 and LUH 5627). The *loci* that encode functions involved in lipopolysaccharide (LPS) biosynthesis have been shown to display important variation across different strains of the same species of animal and plant pathogenic bacteria and this is usually attributed to selection to evade immune responses^32^,^33^,^34^,^35^.

### *n-*alkane metabolism-related genes are conserved

The second stage of *n*-alkane degradation involves the metabolism of such compounds. This is typically carried out through the oxidation of the *n-*alkane corresponding primary alcohol by substrate-specific terminal monooxygenases/hydroxylases. Different classes of enzymes able to carry out this process exist, each of which may confer the capability to degrade compounds with a specific chain length and with a peculiar efficiency. Accordingly, the different hydrocarbon degradation efficiency observed for the *A. venetianus* strains (Fig. 1) may be linked to their repertoire of *n*-alkane degradation related enzymes, besides their emulsifying activity as shown before. To explore this issue, we assembled a dataset of 23 *n*-alkane degradation related protein sequences (Supplementary Table S2) through bibliographical data mining. This included, for example, the complete set of *alk* genes fully characterized in *Pseudomonas putida* GPo1^36^, AlmA from *Acinetobacter* sp. DSM 17874^11^ the soluble cytochrome P450 monooxygenases from *Acinetobacter* sp. EB104^37^. Cytochromes P450, in particular, are heme-thiolate proteins that catalyze the oxygenation of a large number of compounds. Several bacterial strains that degrade C5–C10 alkanes contain alkane hydroxylases that belong to a distinct family of bacterial soluble cytochrome P450 monooxygenases. These cytochromes P450 require the presence of a ferredoxin and of a ferredoxin reductase that transfer electrons from NAD(P)H to the cytochrome^38^.

**Figure 6:**
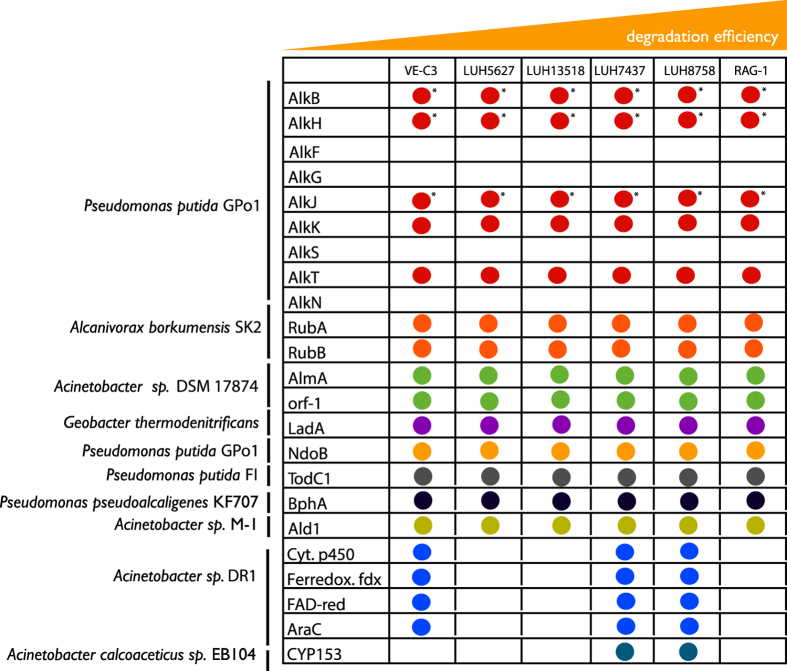
The pattern of presence/absence of genes related to alkane degradation in other species and genera in the six *A. venetianus* strains. An arbitrary degradation efficiency measure is reported on the top of the table, derived from our experimental tests on alkane degradation. The asterisks denote the presence of two homologous copies of the corresponding protein.

These sequences were used as seeds for a BLAST search (with an E-value threshold of 1e-50) on the six *A. venetianus* strains. Results of this analysis are shown in Fig. 6 and revealed that the six genomes harbor an almost complete repertoire of alkane degradation related genes. Notable exceptions to this general trend do exist and include:

1) The absence in all the six *A. venetianus* strains of the genes coding for AlkS, F, G and N proteins. AlkS is the transcriptional regulator of the entire alkane degradation pathway and, in the presence of alkanes, it activates the *alkB* promoter, from which the *alk*BFGHJKL operon is expressed. The absence of this sequence suggests that, in the *A. venetianus* strains analysed so far, alkane degradation pathway is regulated by different mechanisms (see below) or constitutively expressed (as in the case of *Dietzia* sp. H0B^39^ and for specific ubredoxin and rubredoxin reductase in *A. baylyi* ADP1^40^). Recently, a detailed list of the genes involved in the regulation of alkane degradation process has been assembled^41^. We used this information to search for possible alternative regulation mechanisms in *A. venetianus*. More in detail, we searched the six *A. venetianus* strains for another ten putative regulators (see Supplementary Dataset S3) for the complete list of retrieved sequences) and analysed their genomic context for the presence of alkane degradation related genes. Five regulators did not have homologs in any of the *A. venetianus* strains analysed. Out of the remaining five regulators, three did not have any *alk*-like gene in their proximity and we assumed that they were involved in the regulation of some other biological process(es). Conversely, two of them (namely the orthologs of *alkR* and *oruR* from *A. baylyi* ADP1 and *Acinetobacter* sp. M1^40^,^42^, respectively) were both located upstream of the two *alkane 1-monooxygenase* (*alkB* genes) coding genes. Based on this genome-based preliminary exploration, further gene expression studies will be necessary to fully unravel the *alk* genes regulatory network in *A. venetianus.*

No homolog was found for *alkN*, which in *P. putida* GPo1 encodes a methyl-accepting transducer protein, likely involved in chemotaxis towards alkanes in any of the genomes analysed. We also investigated the presence of the *tplS* gene and the chemotaxis machinery described in *Alcanivorax dieselolei* B-5 (encoded by the *che* gene^41^,^43^), but no significant hits were found for these systems in *A. venetianus*. These data raise the intriguing question on the molecular mechanisms used by *A. venetianus* for sensing the presence of *n*-alkanes in the environment. This feature might be of interest given that other *Acinetobacter* species have been shown to possess chemotaxis in response to the presence of alkanes in the medium and that this feature might be used in the context of bioreporter development^44^.

The absence of *alkF* and *G* (Rubredoxin 1 and 2, respectively) in the genomes of *A. venetianus* can be explained by the fact that the function of these genes can be performed by *rubA* and *rubB*, as also shown for *A. baylyi* for which the latter genes have been shown to be essential for the alkane degradation process to be carried out^45^.

2) Three out of six strains (namely VE-C3, LUH 5627 and LUH 7437) encode cytochrome P450 in an operon-like structure with genes encoding a ferredoxin, an FAD-dependent oxyreductase and a gene encoding an AraC transcriptional regulator.

3) Only two strains (namely LUH 8758 and LUH 7437) possess a sequence homologous to CYP153. This sequence has been deeply studied in *Acinetobacter* sp. EB104^46^ and has been shown to be responsible for the hydroxylation of medium-chain-length alkanes^47^. Despite further experimental tests will be needed to infer the actual role of CYP153 in the context of *A. venetianus* alkane degradation, it is tempting to speculate that the presence of this gene could be the cause of the greater efficiency of LUH 7437 and LUH 8758 strains in respect to the other *A. venetianus* strains tested herein (with the exception of RAG1^T^).

## Discussion

In this study, we have exploited a dual approach to more accurately characterize the differences in the alkane degradation process of six *A. venetianus* strains. First, we have phenotypically characterized and compared the six strains, revealing an overall metabolic similarity among them. Differences, however, emerged when the specific *n*-alkane degradation efficiency was tested for each strain, revealing that some strains (and in particular RAG-1^T^) clearly outperform the others. Interestingly, the best *n*-alkane degraders (RAG-1^T^ and LUH 7437), also share the capability to emulsify fuel oil, while this feature is absent in the other strains^24^. To characterize the genetic determinants possibly accounting for such phenotypic diversity, we then sequenced the genomes of these strains and combined comparative genomics with phenotypic data. The results revealed that most likely the presence of a functional and highly specialized emulsan gene cluster is crucial in determining a robust interaction with the fuel oil droplet and in providing a greater efficiency in the overall process of hydrocarbon degradation, being the best two *n*-alkane degraders RAG-1^T^ and LUH 7437 the strains sharing the capability to emulsify oil and the highest conservation of the emulsan biosynthetic gene cluster. Overall, this cluster is poorly conserved from a structural viewpoint among the other strains (despite their phylogenetic relatedness) thus suggesting that several independent events (most likely HGT events) occurred, leading to the current organization of this cluster in these bacteria . The presence of a repertoire of genes known to be involved in *n-*alkane degradation was also checked in the analysed genomes, revealing an overall similarity, except for the presence of specific genes (e.g. the CYP153 protein also found also in *A. calcoaceticus* was only present in LUH 5627 and LUH 7437) that might confer greater efficiency in *n-*alkane degradation to those strains harbouring it. Nevertheless, further experimental tests will be required to validate their exact role and their relevance for bioremediation in these strains.

Overall, this study provided an accurate evaluation of the efficiency of degradation of saturated alkanes present in diesel fuel under one given laboratory condition in *A. venetianus*; moreover, a list of the possible the genetic/genomic features involved in such process was depicted. These genes and (catabolic) pathways represent the most promising set of candidates for future experimental characterization of bioremediation related processes in *A. venetianus*.

## Methods

### Genome sequencing, assembly and annotation

Genomic DNA extraction from *A. venetianus* strains LUH 13518 (formerly A092a), LUH 7437, LUH 5627 and LUH 8758 was carried out as previously described^48^ and genome sequencing was performed through Illumina HiSeq 2000. Illumina reads were first trimmed to eliminate low quality base callings. Trimming was performed adopting the dynamic trimming algorithm embedded in the SolexaQA suite^49^, selecting a Phred score threshold value of 30.

Genome assembly was performed using Abyss v.1.3.5^50^ following the strategy described in^51^ to derive the best assembly. Draft genomes were finally annotated with Prokka (v. 1.11)^52^. The whole genome shotgun projects of *A. venetianus* LUH 5627, LUH 13518, LUH 7437, and LUH 8758 have been deposited at DDBJ/EMBL/GenBank under the accession numbers JRUE00000000, JRHX00000000, JRUK00000000 and JRUJ00000000, respectively.

### Phenotype Microarray (PM) and data analysis

*A. venetianus* LUH 13518 was tested on PM plates PM01–02 (carbon sources) and PM03 (nitrogen sources) as previously described^53^. Briefly, the strain was grown overnight at 30 °C on LB where cells were then picked up with a sterile cotton swab and suspended in 15 ml inoculation fluid (IF-0, Biolog). Cell density was adjusted to 81% transmittance (T) on a Biolog turbidimeter. PM plates were inoculated (100 μl per well) with the cell suspension added with 1% (v/v) Dye Mix E (Biolog). The bacterial suspension used for the inoculation of PM03 was supplemented with the carbon source (20 mM sodium succinate, 2 μM ferric citrate). All the PM plates were incubated at 30 °C in an Omnilog Reader (Biolog) for 48 hours.

PM data of *A. venetianus* VE-C3, RAG-1^T^, LUH 7437, LUH 5627 and LUH 8758 and LUH 13518 were obtained from previously published works and were post-processed and analysed using the Ductape suite^27^.

### Hydrocarbon degradation

To determine the diesel fuel hydrocarbon biodegradation capability, each strain was first cultivated from a glycerol stock culture in LB medium (tryptone 10 g/L, yeast extract 5 g/L, NaCl 5 g/L) at room temperature under orbital shaking (150 rpm), and then transferred (5% v/v) first in saline minimum mineral medium (Na_2_HPO_4_ 2.77 g/L, KH_2_PO_4_ 1.0 g/L, Ca(NO_3_)_2_·4H_2_O 0.03 g/L, (NH_4_)_2_SO_4_ 1.0 g/L, MgSO_4_·7H_2_O 0.2 g/L, iron ammonium citrate 0.01 g/L, NaCl 24 g/L), supplemented with yeast extract (0.02 g/L) and diesel fuel as the primary carbon source at the final concentration of 1.0 g/L^54^. The obtained cultures were used as inoculum (5% v/v) in the HC biodegradation tests in saline medium and diesel fuel at room temperature under orbital shaking (150 rpm). Growth on diesel fuel hydrocarbons was performed in baffled glass bottles closed with a Teflon-coated screw cap, in order to prevent losses of the lighter hydrocarbons fraction by volatilization, and filled with a medium volume of 1/10 of the headspace volume, in order to provide a sufficient amount of oxygen throughout incubation. Duplicate cultures were sacrificed at the beginning of the incubation and after 1, 2, 3, 4 and 7 days of growth to analyze biomass growth and hydrocarbons concentration. Abiotic (uninoculated) controls were also prepared by autoclaving bottles (121 °C, 20 min) containing medium before diesel fuel supplementation. Abiotic controls were sacrificed after 0, 2, 4 and 7 days of incubation in order to quantify possible hydrocarbon abiotic losses.

Before each sampling, sacrificial cultures were incubated at 4 °C for 30 min to reduce light hydrocarbon volatilization; then, hydrocarbons were batch extracted by adding to the culture an equal volume of hexane:acetone (9:1), mixing and sonicating for 10 min. Extraction yield was 96 ± 4%. Hydrocarbon analyses were performed with a 7890 G gas chromatograph (Agilent Technologies Italy S.p.A., Cernusco sul Naviglio, Italy), equipped with an HP-5 capillary column (30 m by 0,25 mm; 0.25 μm) and a flame ionization detector (FID) under the analytical conditions described elsewhere^55^. For the determination of total diesel fuel hydrocarbons (including most unresolved semivolatiles) the overall area of the chromatogram (excluding the solvent peak) was used along with a six point calibration curve obtained by analyzing diesel fuel solutions (in hexane) in the concentration range of 50–1000 mg/L under the same analytical conditions and using the same chromatogram integration approach. *n*-C_10_ to *n-*C_25_ alkanes were identified by comparing retention times with those of an analytical standard *n*-C_10_ to *n-*C_25_ mixture. A six point linear calibration curve in the range 1–50 mg/L was used for the quantification of *n*-C_10_ to *n-*C_25_ alkanes. The strains were ranked according to their biodegradation performances taking into account both the final biodegradation extent (C/C_0_ after 7 days) and the initial degradation rate (C/C_0_ after 1 day) of both total hydrocarbons and of *n*-alkanes. In particular, biodegradation percentages after 1 and 7 days for total hydrocarbons and for *n*-alkanes were summed for each strain to obtain a score; strains were ranked from the lowest score (best performing) to the highest one.

### Chemicals

Diesel fuel was taken from a petroleum gas station as described in^54^. *n*-alkane analytical standards were purchased from ULTRA Scientific Italia s.r.l. (Bologna, Italy), solvents from CARLO ERBA Reagents S.r.l. (Cornaredo, Italy), Chemicals frpm Sigma-Aldrich s.r.l. (Milan, Italy).

### Pangenome construction and genome-scale phylogeny

*A. venetianus* pangenome was constructed using an *ad hoc* developed pipeline (freely available at https://sourceforge.net/projects/parapipe/) exploiting Inparanoid^56^ and Multiparanoid^57^ tools.

To compute whole *Acinetobacter* genus pangenome, a dataset of genome sequences, including i) those used by Touchon *et al*. (2014), ii) the 6 genome sequences described in this work and iii) others that were not included in the Touchon *et al*. (2014) dataset but were available in GenBank, was assembled to obtain a representative dataset for the *Acinetobacter* genus. The final dataset comprised 143 genomes assigned to 33 species (see Supplementary Table S1 for the list of genome accessions). The genomes were annotated with Prokka (v. 1.11)^52^ and used to produce a pangenome with Roary (v. 3.5.7)^58^. The 753 cluster of orthologous proteins retrieved were aligned with Muscle^59^ and misaligned regions were removed where necessary. Finally, all the multialignments were concatenated in a single one (265354 residues) that was used for the phylogenetic analysis. The phylogenetic tree was constructed with Mega6 using the Neighbor joining algorithm, 1000 bootstrap replicates, the Poisson model assuming uniform rates across aligned sites.

## Additional Information

**How to cite this article**: Fondi, M. *et al*. Genomic and phenotypic characterization of the species *Acinetobacter venetianus*. *Sci. Rep.* **6**, 21985; doi: 10.1038/srep21985 (2016).

## Supplementary Material

Supplementary Information

Supplementary Dataset S3

Supplementary Dataset S2

Supplementary Dataset S1

## Figures and Tables

**Table 1:** Main features of the *A. venetianus* genomes analysed in this work.

	LUH 5627	LUH 13518	LUH 7437	LUH 8758	VE-C3	RAG1^T^
Accession number	JRUE00000000	JRHX00000000	JRUK00000000	JRUJ00000000	ALIG00000000	AKIQ00000000
Citation	This work	This work	This work	This work	23	22
Isolation site	Aquaculture pond, Denmark	Dung heap, Germany	Vegetable market, China	Seawater, Japan	Venice lagoon, Italy	Tar on a beach, Israel
Size (nucleotides)	3763378	3537772	3593463	3524791	3564836	3464338
GC content (%) of chromosome	39.23	40.54	40.40	38.31	36.41	39.38
Protein coding genes	3557	3330	3405	3235	3472	3188
Average protein length (amino acids)	304	304	305	311	309	312
Maximum protein length (amino acids)	3489	2334	1873	2206	1797	2052
rRNA operons (16S–23S–5S)	18	15	8	6	6	8
tRNAs	52	60	41	46	74	73
Number of contigs	264	93	90	399	4	87
