# Supplementary material for: Genomic and phenotypic characterization of the species Acinetobacter venetianus
Source: Sci Rep. 2016 Feb 23;6:21985. doi: 10.1038/srep21985 (PMC4763211; doi:10.1038/srep21985)
Supplement: Supplementary Information [file srep21985-s1.pdf]

Supplementary information

Genomic and phenotypic characterization of the species *Acinetobacter venetianus*.

Marco Fondi[1,2], Isabel Maida[1], Elena Perrin[1], Valerio Orlandini[1,2,3], Laura La Torre[1,2], Emanuele Bosi [1,2], Andrea Negroni[4], Giulio Zanaroli[4], Fabio Fava[4], Francesca Decorosi[5], Luciana Giovannetti[5], Carlo Viti[5], Mario Vaneechoutte[6], Lenie Dijkshoorn[7], Renato Fani[1,2]*

[1] Dep. of Biology, ComBo, Florence Computational Biology Group, University of Florence, I-50019 Sesto F.no, Italy,

[2] Dep. of Biology, LEMM, Laboratory of Microbial and Molecular Evolution Florence, University of Florence, I-50019 Sesto F.no, Italy,

[3] Dep. of Clinical and Experimental Biomedical Science "Mario Serio", University of Florence, Viale Pieraccini, 6 - I-50139 Florence

[4]Dep. of Civil, Chemical, Environmental and Materials Engineering (DICAM), University of Bologna, I-40131 Bologna, Italy

[5]Dipartimento di Scienze delle Produzioni Agroalimentari e dell'Ambiente (DISPAA), University of Florence, P.le delle Cascine, 24, Florence, 50144, Italy

[6]Laboratory Bacteriology Research, Faculty Medicine & Health Sciences, University of Ghent, Belgium

7 Dep. of Infectious Diseases, Leiden University Medical Center, PO Box 9600, 2300 RC, Leiden, The Netherlands

* corresponding author:
Prof. Renato Fani , Dep. of Biology, University of Florence
Via Madonna del Piano 6, 50019 Sesto F.no Firenze, Italy
Tel: +39 055 0554574736,
E-mail: renato.fani@unifi.it
http://www.unifi.it/dblemm

Running title: Multi-level exploration of *A. ventianus*

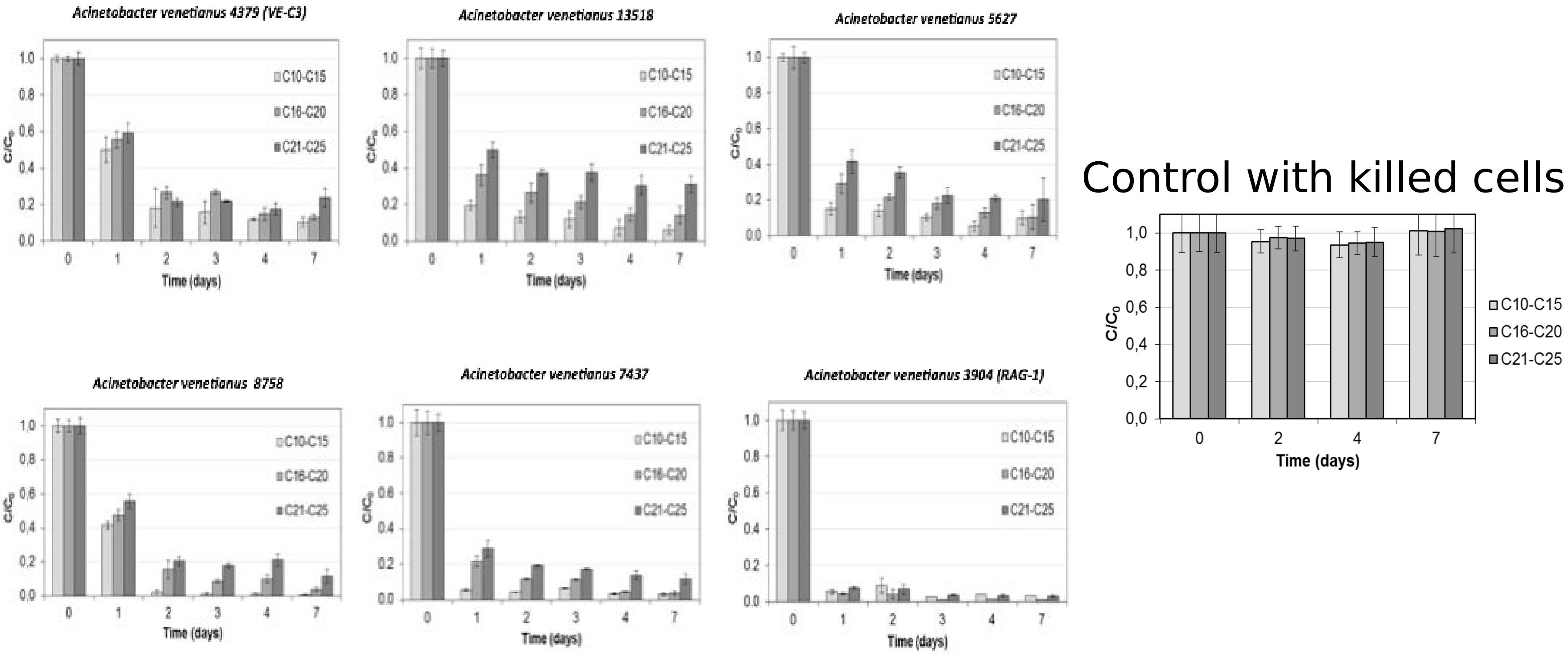

Figure S1: The degradation efficiency for n-alkanes having different molecular weights (MW)

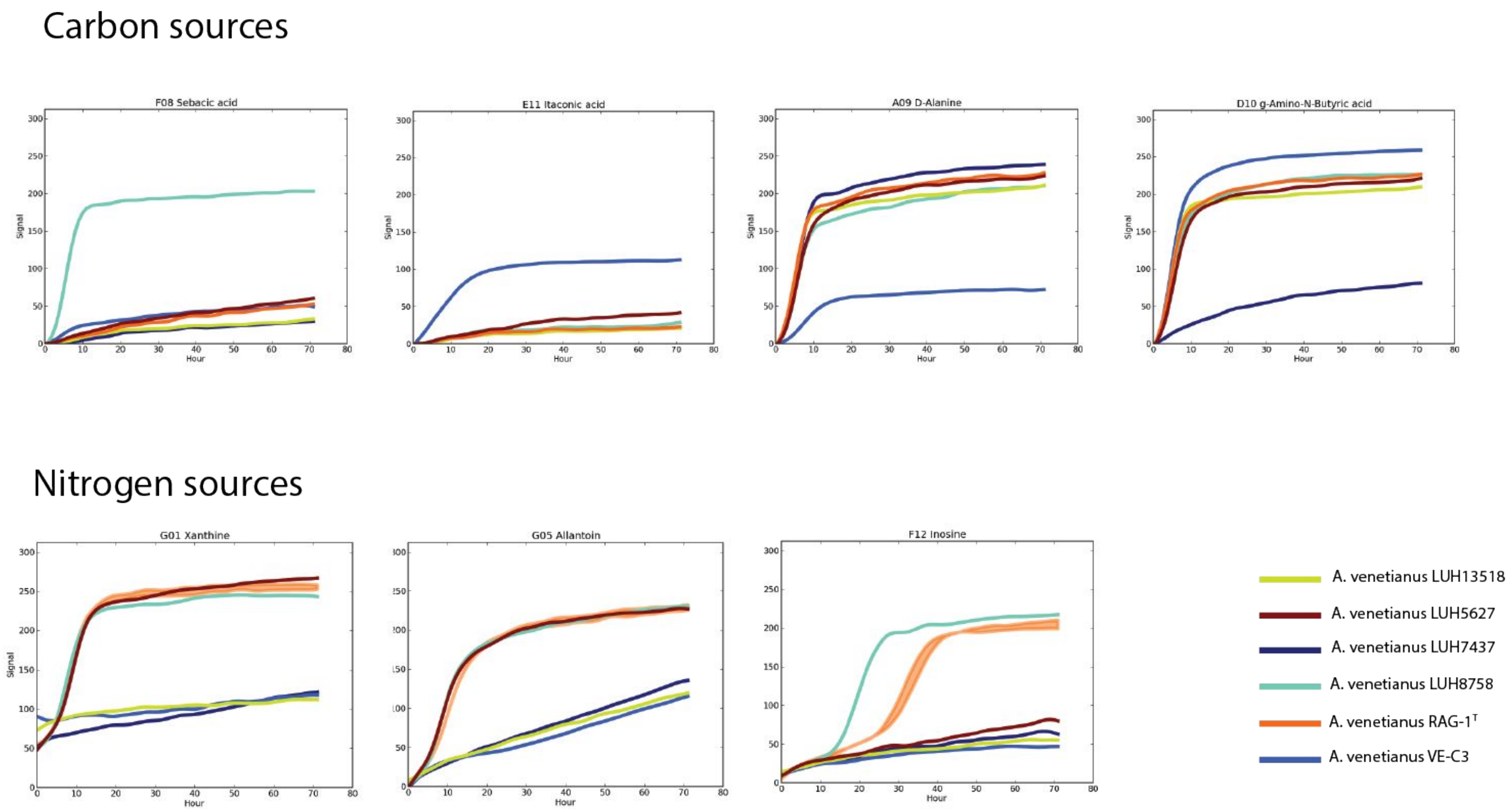

Figure S2: Metabolic capabilities on specific C and N sources of the strains analysed

Genomic and phenotypic characterization of the species Acinetobacter venetianus.

Marco Fondi, Isabel Maida, Elena Perrin, Valerio Orlandini, Laura La Torre, Emanuele Bosi , Andrea Negroni, Giulio Zanaroli, Fabio Fava, Francesca Decorosi, Luciana Giovannetti, Carlo Viti, Mario Vaneechoutte, Lenie Dijkshoorn, Renato Fani

Supplementary Table S1: The list of Acinetobacter genomes analysed

**Accession code**	**Organism name**
GCA_000368985.1	Acinetobacter beijerinckii ANC 3835 (g-proteobacteria)
GCA_000369505.1	Acinetobacter sp. NIPH 298 (g-proteobacteria)
JRHX00000000	A_venetianus_LUH13518
GCA_000368925.1	Acinetobacter bereziniae LMG 1003 = CIP 70.12 (g-proteobacteria)
GCA_000413915.1	Acinetobacter baumannii NIPH 410 (g-proteobacteria)
GCA_000367865.1	Acinetobacter calcoaceticus NIPH 13 (g-proteobacteria)
GCA_000368705.1	Acinetobacter soli CIP 110264 (g-proteobacteria)
GCA_000368665.1	Acinetobacter junii CIP 107470 (g-proteobacteria)
GCA_000369045.1	Acinetobacter pittii ATCC 19004 = CIP 70.29 (g-proteobacteria)
GCA_000368445.1	Acinetobacter sp. CIP 56.2 (g-proteobacteria)
GCA_000369165.1	Acinetobacter baumannii NIPH 201 (g-proteobacteria)
GCA_000369265.1	Acinetobacter baumannii NIPH 67 (g-proteobacteria)
GCA_000368245.1	Acinetobacter baumannii NIPH 1734 (g-proteobacteria)
GCA_000368685.1	Acinetobacter baylyi DSM 14961 = CIP 107474 (g-proteobacteria)
GCA_000368945.1	Acinetobacter calcoaceticus ANC 3680 (g-proteobacteria)
GCA_000367965.1	Acinetobacter sp. NIPH 236 (g-proteobacteria)
GCA_000369025.1	Acinetobacter pittii ANC 3678 (g-proteobacteria)
GCA_000369805.1	Acinetobacter sp. NIPH 2100 (g-proteobacteria)
GCA_000368605.1	Acinetobacter nosocomialis NIPH 386 (g-proteobacteria)
GCA_000400735.1	Acinetobacter tandoii DSM 14970 = CIP 107469 (g-proteobacteria)
GCA_000369385.1	Acinetobacter baumannii ATCC 19606 = CIP 70.34 (g-proteobacteria)
GCA_000369345.1	Acinetobacter baumannii NIPH 80 (g-proteobacteria)
GCA_000369305.1	Acinetobacter baumannii NIPH 70 (g-proteobacteria)
GCA_000803285.1	Acinetobacter oleivorans PF1 (g-proteobacteria)
GCA_000369785.1	Acinetobacter sp. NIPH 3623 (g-proteobacteria)
GCA_000369465.1	Acinetobacter sp. CIP 53.82 (g-proteobacteria)
GCA_000369145.1	Acinetobacter lwoffii NIPH 478 (g-proteobacteria)
GCA_000368105.1	Acinetobacter baumannii NIPH 1669 (g-proteobacteria)
GCA_000368205.1	Acinetobacter baumannii NIPH 615 (g-proteobacteria)
GCA_000188215.1	Acinetobacter baumannii 1656-2 (g-proteobacteria)
GCA_000368325.1	Acinetobacter sp. CIP 102529 (g-proteobacteria)
GCA_000368825.1	Acinetobacter ursingii DSM 16037 = CIP 107286 (g-proteobacteria)
GCA_000368625.1	Acinetobacter schindleri CIP 107287 (g-proteobacteria)
GCA_000488235.1	Acinetobacter oleivorans CIP 110421 (g-proteobacteria)
GCA_000368745.1	Acinetobacter junii NIPH 182 (g-proteobacteria)
GCA_000731975.1	Acinetobacter baumannii MRSN 3405 (g-proteobacteria)
GCA_000368405.1	Acinetobacter sp. NIPH 817 (g-proteobacteria)
GCA_000368185.1	Acinetobacter baumannii NIPH 146 (g-proteobacteria)
GCA_000368005.1	Acinetobacter parvus NIPH 1103 (g-proteobacteria)
GCA_000413855.1	Acinetobacter gyllenbergii CIP 110306 (g-proteobacteria)
GCA_000369865.1	Acinetobacter sp. CIP 102143 (g-proteobacteria)
GCA_000369105.1	Acinetobacter lwoffii NCTC 5866 = CIP 64.10 = NIPH 512 (g-proteobacteria)
GCA_000369885.1	Acinetobacter ursingii NIPH 706 (g-proteobacteria)
GCA_000368165.1	Acinetobacter lwoffii NIPH 715 (g-proteobacteria)
GCA_000413875.1	Acinetobacter indicus ANC 4215 (g-proteobacteria)
GCA_000400715.1	Acinetobacter sp. CIP 110321 (g-proteobacteria)
GCA_000368285.1	Acinetobacter sp. CIP 102159 (g-proteobacteria)
GCA_000189735.2	Acinetobacter baumannii TCDC-AB0715 (g-proteobacteria)
GCA_000369605.1	Acinetobacter sp. NIPH 1847 (g-proteobacteria)

GCA_000369625.1	Acinetobacter sp. NIPH 2171 (g-proteobacteria)
GCA_000369065.1	Acinetobacter haemolyticus CIP 64.3 (g-proteobacteria)
AKIQ00000000	A_venetianus_RAG1T
GCA_000368065.1	Acinetobacter seifertii NIPH 973 (g-proteobacteria)
GCA_000368645.1	Acinetobacter brisouii ANC 4119 (g-proteobacteria)
GCA_000368585.1	Acinetobacter venetianus RAG-1 = CIP 110063 (g-proteobacteria)
GCA_000367925.1	Acinetobacter bohemicus ANC 3994 (g-proteobacteria)
GCA_000413895.1	Acinetobacter rudis CIP 110305 (g-proteobacteria)
GCA_000369845.1	Acinetobacter sp. ANC 3880 (g-proteobacteria)
GCA_000368265.1	Acinetobacter sp. ANC 3789 (g-proteobacteria)
GCA_000069205.1	Acinetobacter baumannii SDF (g-proteobacteria)
GCA_000487975.1	Acinetobacter lwoffii NCTC 5866 = CIP 64.10 = NIPH 512 (g-proteobacteria)
GCA_000368545.1	Acinetobacter baumannii NIPH 60 (g-proteobacteria)
GCA_000368485.1	Acinetobacter guillouiae NIPH 991 (g-proteobacteria)
GCA_000368885.1	Acinetobacter radioresistens NIPH 2130 (g-proteobacteria)
GCA_000368725.1	Acinetobacter soli NIPH 2899 (g-proteobacteria)
JRUE00000000	A_venetianus_LUH5627
GCA_000488215.1	Acinetobacter nectaris CIP 110549 (g-proteobacteria)
GCA_000369525.1	Acinetobacter sp. CIP 70.18 (g-proteobacteria)
GCA_000413935.1	Acinetobacter sp. NIPH 2036 (g-proteobacteria)
GCA_000369085.1	Acinetobacter haemolyticus NIPH 261 (g-proteobacteria)
GCA_000196795.1	Acinetobacter oleivorans DR1 (g-proteobacteria)
GCA_000368085.1	Acinetobacter nosocomialis NIPH 2119 (g-proteobacteria)
GCA_000368845.1	Acinetobacter ursingii ANC 3649 (g-proteobacteria)
GCA_000369365.1	Acinetobacter baumannii ANC 4097 (g-proteobacteria)
GCA_000369205.1	Acinetobacter baumannii NIPH 335 (g-proteobacteria)
GCA_000369685.1	Acinetobacter sp. CIP 102136 (g-proteobacteria)
GCA_000369705.1	Acinetobacter sp. NIPH 2168 (g-proteobacteria)
GCA_000368225.1	Acinetobacter baumannii NIPH 2061 (g-proteobacteria)
GCA_000021245.1	Acinetobacter baumannii AB0057 (g-proteobacteria)
GCA_000191145.1	Acinetobacter pittii PHEA-2 (g-proteobacteria)
GCA_000399665.1	Acinetobacter calcoaceticus ANC 3811 (g-proteobacteria)
GCA_000368865.1	Acinetobacter bouvetii DSM 14964 = CIP 107468 (g-proteobacteria)
GCA_000369245.1	Acinetobacter baumannii NIPH 601 (g-proteobacteria)
GCA_000368345.1	Acinetobacter sp. NIPH 758 (g-proteobacteria)
GCA_000368765.1	Acinetobacter junii CIP 64.5 (g-proteobacteria)
GCA_000368805.1	Acinetobacter johnsonii ANC 3681 (g-proteobacteria)
GCA_000369565.1	Acinetobacter sp. ANC 3862 (g-proteobacteria)
GCA_000369665.1	Acinetobacter sp. CIP 51.11 (g-proteobacteria)
GCA_000367885.1	Acinetobacter baumannii NIPH 24 (g-proteobacteria)
GCA_000069245.1	Acinetobacter baumannii AYE (g-proteobacteria)
GCA_001307195.1	Acinetobacter equi 114 (g-proteobacteria)
GCA_000046845.1	Acinetobacter sp. ADP1 (g-proteobacteria)
GCA_000369745.1	Acinetobacter sp. CIP 64.7 (g-proteobacteria)
GCA_000368785.1	Acinetobacter towneri DSM 14962 = CIP 107472 (g-proteobacteria)
GCA_000731965.1	Acinetobacter baumannii (g-proteobacteria) BM4587
GCA_000836035.1	Acinetobacter oleivorans KCTC 23045 (g-proteobacteria)
GCA_000369325.1	Acinetobacter baumannii NIPH 290 (g-proteobacteria)
GCA_000368425.1	Acinetobacter sp. CIP 102637 (g-proteobacteria)
GCA_000368525.1	Acinetobacter baumannii NIPH 190 (g-proteobacteria)
GCA_000226275.1	Acinetobacter baumannii MDR-ZJ06 (g-proteobacteria)
GCA_000367985.1	Acinetobacter sp. CIP-A165 (g-proteobacteria)
GCA_000368385.1	Acinetobacter sp. NIPH 899 (g-proteobacteria)
GCA_000816495.1	Acinetobacter harbinensis HITLi 7 (g-proteobacteria)
GCA_000368965.1	Acinetobacter calcoaceticus DSM 30006 = CIP 81.8 (g-proteobacteria)
GCA_000368305.1	Acinetobacter sp. CIP 102129 (g-proteobacteria)
GCA_000367905.1	Acinetobacter sp. CIP A162 (g-proteobacteria)
GCA_000368505.1	Acinetobacter bereziniae NIPH 3 (g-proteobacteria)

GCA_000368565.1	Acinetobacter gerneri DSM 14967 = CIP 107464 (g-proteobacteria)
GCA_000369285.1	Acinetobacter baumannii NIPH 528 (g-proteobacteria)
GCA_000369445.1	Acinetobacter sp. NIPH 713 (g-proteobacteria)
GCA_000368125.1	Acinetobacter baumannii NIPH 1362 (g-proteobacteria)
GCA_000369485.1	Acinetobacter sp. ANC 4105 (g-proteobacteria)
GCA_000369425.1	Acinetobacter sp. NIPH 284 (g-proteobacteria)
GCA_000369585.1	Acinetobacter sp. CIP 101934 (g-proteobacteria)
GCA_000367945.1	Acinetobacter sp. NIPH 809 (g-proteobacteria)
GCA_000369765.1	Acinetobacter sp. NIPH 1859 (g-proteobacteria)
GCA_000369825.1	Acinetobacter sp. NIPH 542 (g-proteobacteria)
GCA_000369545.1	Acinetobacter sp. NIPH 1867 (g-proteobacteria)
GCA_000515995.2	Acinetobacter baumannii UH9007 (g-proteobacteria)
GCA_000488195.1	Acinetobacter gyllenbergii NIPH 230 (g-proteobacteria)
JRUK00000000	A_venetianus_LUH7437
GCA_000015425.1	Acinetobacter baumannii ATCC 17978 (g-proteobacteria)
GCA_000369185.1	Acinetobacter baumannii NIPH 527 (g-proteobacteria)
GCA_000187205.4	Acinetobacter baumannii MDR-TJ (g-proteobacteria)
GCA_000368045.1	Acinetobacter johnsonii CIP 64.6 (g-proteobacteria)
GCA_000488175.1	Acinetobacter tjernbergiae DSM 14971 = CIP 107465 (g-proteobacteria)
JRUJ00000000	A_venetianus_LUH8758
GCA_000368465.1	Acinetobacter schindleri NIPH 900 (g-proteobacteria)
GCA_000369405.1	Acinetobacter sp. ANC 3929 (g-proteobacteria)
GCA_000488255.1	Acinetobacter indicus CIP 110367 (g-proteobacteria)
GCA_000368025.1	Acinetobacter parvus DSM 16617 = CIP 108168 (g-proteobacteria)
GCA_000368365.1	Acinetobacter sp. CIP 102082 (g-proteobacteria)
GCA_000369725.1	Acinetobacter sp. CIP 101966 (g-proteobacteria)
GCA_000399705.1	Acinetobacter pittii ANC 4052 (g-proteobacteria)
GCA_000369225.1	Acinetobacter baumannii NIPH 329 (g-proteobacteria)
GCA_000369005.1	Acinetobacter beijerinckii CIP 110307 (g-proteobacteria)
ALIG00000000	A_venetianus_VEC3
GCA_000399685.1	Acinetobacter pittii ANC 4050 (g-proteobacteria)
GCA_000369645.1	Acinetobacter sp. CIP 64.2 (g-proteobacteria)
GCA_000302575.1	Acinetobacter baumannii TYTH-1 (g-proteobacteria)
GCA_000368905.1	Acinetobacter radioresistens DSM 6976 = NBRC 102413 = CIP 103788 (g-proteobacteria)
GCA_000368145.1	Acinetobacter guillouiae CIP 63.46 (g-proteobacteria)
GCA_000021145.1	Acinetobacter baumannii AB307-0294 (g-proteobacteria)

Gene	Enzyme class	Substrate	Presence shown in:	Accession *	References
nonM	*Oxygenase systems P450 CYP153*<br>*- P450 oxygenase: P450 heme*<br>*- Ferredoxin: iron-sulfur*<br>*- Ferredoxin reductase: FAD, NADH*	*C4-C16 n-alkanes, cycloalkanes*	*Acinetobacter calcoaceticus sp. EB104*	*gb: NP114222*	*[49]*
alkM	*Alkane-1-monoxygenase*	*C7-C18 n-alkanes*	*Acinetobacter sp. ADP-1*	*gb: YP046098*	[50]
almA	*Alm: flavin binding monooxygenase*	*>C32 alkanes*	*Acinetobacter sp.DSM17874*	*gb: ABQ18224*	[51]
ladA	*Long-chain alkane monoxygenase*	*>C15 n-alkanes*	*Geobacillus thermodenitrificans NG80-2*	*gb: YP001127577*	[52]
ndoB	*α-subunit of Naphthalene 1,2-dioxygenase*	*aromatic compound*	*Pseudomonas putida*	*gb: P0A110*	[53]
todC1	*α-subunit of terminal toluene 2,3-dioxygenase*	*aromatic compound*	*P. putida F1*	*gb: A5W4F2*	[54]
bphA	*α-subunit of Biphenyl dioxygenase*	*aromatic compound*	*P. pseudoalcaligenes KF707*	*gb: Q52028*	[55]
ald1	*aldehyde dehydrogenase 1*	*C16-C22 n-alkanes*	*Acinetobacter sp. M-1*	*sp: Q9FDS1*	[56]
	Alkane hydroxylase systems	*C5-C16 n-alkanes*			
alkB	*Alkane-1-monoxygenase*		*Acinetobacter sp. M-1*	*gb: Q9WWW6*	*[14]*
alkF	*Rubredoxin-1*		*P. putida GPo1*	*sp: P12692*	*[14]*
alkG	*Rubredoxin-2*		*P. putida GPo1*	*sp: P00272*	*[14]*
alk H	*Aldehyde dehydrogenase*		*P. putida GPo1*	*sp: P12693*	*[14]*
alk J	*Alcohol dehydrogenase*		*P. putida GPo1*	*sp: Q00593*	*[14]*
alk K	*Medium-chain acyl-CoA synthetase*		*P. putida GPo1*	*sp: Q00594*	*[14]*
alk L	*Outer membrane protein AlkL*		*P. putida GPo1*	*sp: Q00595*	*[14]*
alk S	*Regulator of alkBFGHJKL expression*		*P. putida GPo1*	*sp:P17051*	*[14]*
alk N	*chemotactic transducer*		*P. aeruginosa*	*gb:BAA23413*	*[14]*

Supplementary Table S2: The assembled dataset of 23 n-alkane degradation related protein sequences

[1] J. C. Spain, "Environmental biotechnology research: an overview.," *Aviation, space, and environmental medicine*, vol. 65, no. 5 Suppl, pp. A131-7, May 1994.

[2] R. B. Meagher, "Phytoremediation of toxic elemental and organic pollutants.," *Current opinion in plant biology*, vol. 3, no. 2, pp. 153-62, Apr. 2000.

[3] M. Megharaj, B. Ramakrishnan, K. Venkateswarlu, N. Sethunathan, and R. Naidu, "Bioremediation approaches for organic pollutants: a critical perspective.," *Environment international*, vol. 37, no. 8, pp. 1362-75, Nov. 2011.

[4] H. A. Kulikova, O. I. Kliaĭn, E. V. Stepanova, and O. V. Koroleva, “Use of basidiomycetes in industrial waste processing and utilization technologies: fundamental and applied aspects (review).,” *Prikladnaia biokhimiia i mikrobiologiia*, vol. 47, no. 6, pp. 619-34, 2011.

[5] T. H. Lee, I. G. Byun, Y. O. Kim, I. S. Hwang, and T. J. Park, “Monitoring biodegradation of diesel fuel in bioventing processes using in situ respiration rate.,” *Water science and technology : a journal of the International Association on Water Pollution Research*, vol. 53, no. 4–5, pp. 263-72, Jan. 2006.

[6] M. M. Yakimov et al., “Alcanivorax borkumensis gen. nov., sp. nov., a new, hydrocarbon-degrading and surfactant-producing marine bacterium.,” *International journal of systematic bacteriology*, vol. 48 Pt 2, pp. 339-48, Apr. 1998.

[7] P. N. Golyshin, T. N. Chernikova, W.-R. Abraham, H. Lünsdorf, K. N. Timmis, and M. M. Yakimov, “Oleiphilaceae fam. nov., to include Oleiphilus messinensis gen. nov., sp. nov., a novel marine bacterium that obligately utilizes hydrocarbons.,” *International journal of systematic and evolutionary microbiology*, vol. 52, no. Pt 3, pp. 901-11, May 2002.

[8] J. G. Leahy and R. R. Colwell, “Microbial degradation of hydrocarbons in the environment.,” *Microbiol. Mol. Biol. Rev.*, vol. 54, no. 3, pp. 305-315, Sep. 1990.

[9] M. M. Yakimov et al., “Thalassolituus oleivorans gen. nov., sp. nov., a novel marine bacterium that obligately utilizes hydrocarbons.,” *International journal of systematic and evolutionary microbiology*, vol. 54, no. Pt 1, pp. 141-8, Jan. 2004.

[10] M. M. Yakimov et al., “Oleispira antarctica gen. nov., sp. nov., a novel hydrocarbonoclastic marine bacterium isolated from Antarctic coastal sea water.,” *International journal of systematic and evolutionary microbiology*, vol. 53, no. Pt 3, pp. 779-85, May 2003.

[11] M. Throne-Holst, S. Markussen, A. Winnberg, T. E. Ellingsen, H.-K. Kotlar, and S. B. Zotchev, “Utilization of n-alkanes by a newly isolated strain of Acinetobacter venetianus: the role of two AlkB-type alkane hydroxylases.,” *Applied microbiology and biotechnology*, vol. 72, no. 2, pp. 353-60, Sep. 2006.

[12] A. Wentzel, T. E. Ellingsen, H.-K. Kotlar, S. B. Zotchev, and M. Throne-Holst, “Bacterial metabolism of long-chain n-alkanes.,” *Applied microbiology and biotechnology*, vol. 76, no. 6, pp. 1209-21, Oct. 2007.

[13] J. B. van Beilen and E. G. Funhoff, “Alkane hydroxylases involved in microbial alkane degradation.,” *Applied microbiology and biotechnology*, vol. 74, no. 1, pp. 13-21, Feb. 2007.

[14] J. B. van Beilen, S. Panke, S. Lucchini, A. G. Franchini, M. Röthlisberger, and B. Witholt, “Analysis of Pseudomonas putida alkane-degradation gene clusters and flanking insertion sequences: evolution and regulation of the alk genes.,” *Microbiology (Reading, England)*, vol. 147, no. Pt 6, pp. 1621-30, Jun. 2001.

[15] U. Gerischer, Ed., *Acinetobacter: Molecular Biology*. Norfolk, UK: Caister Academic Press, 2008, p. 358.

[16] F. Di Cello, M. Pepi, F. Baldi, and R. Fani, “Molecular characterization of an n-alkane-degrading bacterial community and identification of a new species, Acinetobacter venetianus.,” *Research in microbiology*, vol. 148, no. 3, pp. 237-49, 1997.

[17] M. Vaneechoutte et al., “Description of Acinetobacter venetianus ex Di Cello et al. 1997 sp. nov.,” *International journal of systematic and evolutionary microbiology*, vol. 59, no. Pt 6, pp. 1376-81, Jun. 2009.

[18] M. Pepi, A. Minacci, F. Di Cello, F. Baldi, and R. Fani, “Long-term analysis of diesel fuel consumption in a co-culture of Acinetobacter venetianus, Pseudomonas putida and Alcaligenes faecalis.,” *Antonie van Leeuwenhoek*, vol. 83, no. 1, pp. 3-9, Jan. 2003.

[19] A. Reisfeld, E. Rosenberg, and D. Gutnick, “Microbial degradation of crude oil: factors affecting the dispersion in sea water by mixed and pure cultures.,” *Applied microbiology*, vol. 24, no. 3, pp. 363-8, Sep. 1972.

[20] M. Vaneechoutte et al., “Oil-degrading Acinetobacter strain RAG-1 and strains described as ‘Acinetobacter venetianus sp. nov.’ belong to the same genomic species,” *Research in Microbiology*, vol. 150, no. 1, pp. 69-73, Jan. 1999.

[21] H. Dams-Kozlowska, M. P. Mercaldi, B. J. Panilaitis, and D. L. Kaplan, “Modifications and applications of the Acinetobacter venetianus RAG-1 exopolysaccharide, the emulsan complex and its components.,” *Applied microbiology and biotechnology*, vol. 81, no. 2, pp. 201-10, Nov. 2008.

[22] G. Amoabediny et al., “Application of a novel method for optimization of bioemulsan production in a miniaturized bioreactor.,” *Bioresource technology*, vol. 101, no. 24, pp. 9758-64, Dec. 2010.

[23] K. Mara et al., “Molecular and phenotypic characterization of Acinetobacter strains able to degrade diesel fuel.,” *Research in microbiology*, vol. 163, no. 3, pp. 161-72, Apr. 2012.

[24] M. P. Mercaldi, H. Dams-Kozlowska, B. Panilaitis, A. P. Joyce, and D. L. Kaplan, “Discovery of the dual polysaccharide composition of emulsan and the isolation of the emulsion stabilizing component.,” *Biomacromolecules*, vol. 9, no. 7, pp. 1988-96, Jul. 2008.

[25] D. Nakar and D. L. Gutnick, “Analysis of the wee gene cluster responsible for the biosynthesis of the polymeric bioemulsifier from the oil-degrading strain Acinetobacter lwoffii RAG-1.,” *Microbiology (Reading, England)*, vol. 147, no. Pt 7, pp. 1937-46, Jul. 2001.

[26] D. Medini, C. Donati, H. Tettelin, V. Masignani, and R. Rappuoli, “The microbial pan-genome.,” *Current opinion in genetics & development*, vol. 15, no. 6, pp. 589-94, Dec. 2005.

[27] S. F. Altschul, W. Gish, W. Miller, E. W. Myers, and D. J. Lipman, “Basic local alignment search tool.,” *Journal of molecular biology*, vol. 215, no. 3, pp. 403-10, Oct. 1990.

[28] “BioEdit Sequence Alignment Editor for Windows 95/98/NT/XP/Vista/7.” [Online]. Available: http://www.mbio.ncsu.edu/bioedit/bioedit.html.

[29] J. D. Thompson, D. G. Higgins, and T. J. Gibson, “CLUSTAL W: improving the sensitivity of progressive multiple sequence alignment through sequence weighting, position-specific gap penalties and weight matrix choice.,” *Nucleic acids research*, vol. 22, no. 22, pp. 4673-80, Nov. 1994.

[30] M. a Larkin et al., “Clustal W and Clustal X version 2.0.,” *Bioinformatics (Oxford, England)*, vol. 23, no. 21, pp. 2947-8, Nov. 2007.

[31] K. Tamura, D. Peterson, N. Peterson, G. Stecher, M. Nei, and S. Kumar, “MEGA5: molecular evolutionary genetics analysis using maximum likelihood, evolutionary distance, and maximum parsimony methods.,” *Molecular biology and evolution*, vol. 28, no. 10, pp. 2731-9, Oct. 2011.

[32] B. R. Bochner, P. Gadzinski, and E. Panomitros, “Phenotype microarrays for high-throughput phenotypic testing and assay of gene function.,” *Genome research*, vol. 11, no. 7, pp. 1246-55, Jul. 2001.

[33] M. Galardini et al., “DuctApe : a suite for the analysis and correlation of genomes and Omnilog TM Phenotype Microarray data,” *(Submitted for publications)*.

[34] “DuctApe, genomics and phenomics made easy.” [Online]. Available: http://combogenomics.github.io/DuctApe/.

[35] T. J. Carver, K. M. Rutherford, M. Berriman, M.-A. Rajandream, B. G. Barrell, and J. Parkhill, “ACT: the Artemis Comparison Tool.,” *Bioinformatics (Oxford, England)*, vol. 21, no. 16, pp. 3422-3, Aug. 2005.

[36] P. Baumann, M. Doudoroff, and R. Y. Stanier, “A study of the Moraxella group. II. Oxidative-negative species (genus Acinetobacter).,” *Journal of bacteriology*, vol. 95, no. 5, pp. 1520-41, May 1968.

[37] A. Nemec et al., “Genotypic and phenotypic characterization of the Acinetobacter calcoaceticus-Acinetobacter baumannii complex with the proposal of Acinetobacter pittii sp. nov. (formerly Acinetobacter genomic species 3) and Acinetobacter nosocomialis sp. nov. (formerly Ac,” *Research in microbiology*, vol. 162, no. 4, pp. 393-404, May 2011.

[38] Y.-C. Chuang et al., “Influence of genospecies of Acinetobacter baumannii complex on clinical outcomes of patients with acinetobacter bacteremia.,” *Clinical infectious diseases : an official publication of the Infectious Diseases Society of America*, vol. 52, no. 3, pp. 352-60, Feb. 2011.

[39] T. H. Koh et al., “Acinetobacter calcoaceticus-Acinetobacter baumannii complex species in clinical specimens in Singapore.,” *Epidemiology and infection*, vol. 140, no. 3, pp. 535-8, Mar. 2012.

[40] J. F. Turton, J. Shah, C. Ozongwu, and R. Pike, “Incidence of Acinetobacter species other than A. baumannii among clinical isolates of Acinetobacter: evidence for emerging species.,” *Journal of clinical microbiology*, vol. 48, no. 4, pp. 1445-9, Apr. 2010.

[41] P. J. van den Broek, T. J. K. van der Reijden, E. van Strijen, A. V. Helmig-Schurter, A. T. Bernards, and L. Dijkshoorn, “Endemic and epidemic acinetobacter species in a university hospital: an 8-year survey.,” *Journal of clinical microbiology*, vol. 47, no. 11, pp. 3593-9, Nov. 2009.

[42] J. H. Yum et al., “Molecular characterization of metallo-beta-lactamase-producing Acinetobacter baumannii and Acinetobacter genomospecies 3 from Korea: identification of two new integrons carrying the bla(VIM-2) gene cassettes.,” *The Journal of antimicrobial chemotherapy*, vol. 49, no. 5, pp. 837-40, May 2002.

[43] M.-C. Chiang et al., “Clinical characteristics and outcomes of bacteremia due to different genomic species of Acinetobacter baumannii complex in patients with solid tumors.,” *Infection*, vol. 40, no. 1, pp. 19-26, Feb. 2012.

[44] J. BRISOU and A. R. PREVOT, “Studies on bacterial taxonomy. X. The revision of species under Acromobacter group.,” *Annales de l’Institut Pasteur*, vol. 86, no. 6, pp. 722-8, Jun. 1954.

[45] A. Y. Peleg, H. Seifert, and D. L. Paterson, “Acinetobacter baumannii: emergence of a successful pathogen.,” *Clinical microbiology reviews*, vol. 21, no. 3, pp. 538-82, Jul. 2008.

[46] A. Ibrahim, P. Gerner-Smidt, and W. Liesack, “Phylogenetic relationship of the twenty-one DNA groups of the genus Acinetobacter as revealed by 16S ribosomal DNA sequence analysis.,” *International journal of systematic bacteriology*, vol. 47, no. 3, pp. 837-41, Jul. 1997.

[47] G. E. Fox, J. D. Wisotzkey, and P. Jurtshuk, “How close is close: 16S rRNA sequence identity may not be sufficient to guarantee species identity.,” *International journal of systematic bacteriology*, vol. 42, no. 1, pp. 166-70, Jan. 1992.

[48] J. Z.-M. Chan, M. R. Halachev, N. J. Loman, C. Constantinidou, and M. J. Pallen, “Defining bacterial species in the genomic era: insights from the genus Acinetobacter.,” *BMC microbiology*, vol. 12, p. 302, Jan. 2012.

[49] T. Maier, H. H. Förster, O. Asperger, and U. Hahn, “Molecular characterization of the 56-kDa CYP153 from Acinetobacter sp. EB104.,” *Biochemical and biophysical research communications*, vol. 286, no. 3, pp. 652-8, Aug. 2001.

[50] A. Ratajczak, W. Geissdörfer, and W. Hillen, “Expression of alkane hydroxylase from Acinetobacter sp. Strain ADP1 is induced by a broad range of n-alkanes and requires the transcriptional activator AlkR.,” *Journal of bacteriology*, vol. 180, no. 22, pp. 5822-7, Nov. 1998.

[51] M. Throne-Holst, A. Wentzel, T. E. Ellingsen, H.-K. Kotlar, and S. B. Zotchev, “Identification of novel genes involved in long-chain n-alkane degradation by Acinetobacter sp. strain DSM 17874.,” *Applied and environmental microbiology*, vol. 73, no. 10, pp. 3327-32, May 2007.

[52] L. Feng et al., “Genome and proteome of long-chain alkane degrading Geobacillus thermodenitrificans NG80-2 isolated from a deep-subsurface oil reservoir,” *Proceedings of the National Academy of Sciences*, vol. 104, no. 13, pp. 5602-5607, Mar. 2007.

[53] S. Kurkela, H. Lehväslaiho, E. T. Palva, and T. H. Teeri, “Cloning, nucleotide sequence and characterization of genes encoding naphthalene dioxygenase of Pseudomonas putida strain NCIB9816,” *Gene*, vol. 73, no. 2, pp. 355-362, Dec. 1988.

[54] G. Zylstra and D. Gibson, “Toluene degradation by Pseudomonas putida F1. Nucleotide sequence of the todC1C2BADE genes and their expression in Escherichia coli,” *J. Biol. Chem.*, vol. 264, no. 25, pp. 14940-14946, Sep. 1989.

[55] K. Furukawa, N. Arimura, and T. Miyazaki, “Nucleotide sequence of the 2,3-dihydroxybiphenyl dioxygenase gene of Pseudomonas pseudoalcaligenes.,” *Journal of bacteriology*, vol. 169, no. 1, pp. 427-9, Jan. 1987.

[56] T. Ishige, a Tani, Y. Sakai, and N. Kato, “Long-chain aldehyde dehydrogenase that participates in n-alkane utilization and wax ester synthesis in Acinetobacter sp. strain M-1.,” *Applied and environmental microbiology*, vol. 66, no. 8, pp. 3481-6, Aug. 2000.

[57] J. B. van Beilen et al., “Cytochrome P450 alkane hydroxylases of the CYP153 family are common in alkane-degrading eubacteria lacking integral membrane alkane hydroxylases.,” *Applied and environmental microbiology*, vol. 72, no. 1, pp. 59-65, Jan. 2006.
